# Failure of Split Posterior Tibial Tendon Transfer in Cerebral Palsy Complex Foot Deformities: A Review of Failure Definitions and Risk Factors for Failure

**DOI:** 10.1007/s12178-025-09975-6

**Published:** 2025-05-03

**Authors:** Hussein Hashem, Joseph Hayek, Hassan Abou Adma, Karim Gaber, Waleed Kishta

**Affiliations:** 1 Division of Orthopaedic Surgery, Department of Surgery, McMaster University, 1200 Main St W, Room 4E15, Hamilton, ON L8N 3Z5 Canada; 2 School of Medicine, University of Galway, Galway, Ireland; 3 Saint James School of Medicine, Caribbean Island, Anguilla

**Keywords:** Cerebral palsy, Spastic equinovarus deformity, Split posterior tibial tendon transfer (SPOTT), Surgical failure, Recurrence, Pediatric foot deformity

## Abstract

**Purpose of Review:**

This review examines variability in failure and recurrence rates following split posterior tibial tendon transfer (SPOTT) for spastic equinovarus deformity (SED) in children with cerebral palsy (CP). It evaluates patient-specific and surgical risk factors contributing to poor outcomes and assesses the inconsistent definitions of failure across the literature.

**Recent Findings:**

Across the seven included studies, failure was more common in patients under the age of 8, non-ambulatory individuals, and those with quadriplegic CP. Surgical contributors included poor tendon tensioning, residual spasticity, over- or under-correction, and untreated bony deformities. Although modified techniques demonstrated improved outcomes, the risk of recurrence was not completely eliminated. All studies consistently lacked standardized definitions of surgical failure and recurrence, limiting cross-study comparability. Postoperative management strategies—particularly structured bracing protocols and delaying surgery until after age 8—were associated with more favorable outcomes.

**Summary:**

SPOTT remains a viable surgical option for dynamic SED in children with CP, but long-term success is highly dependent on careful patient selection, surgical expertise, and consistent postoperative care. Inconsistent definitions of recurrence and failure remain a major barrier to improving clinical outcomes and conducting meaningful comparative research. To enhance clinical decision-making and guide future studies, a standardized grading system should be urgently developed and adopted in the field.

## Introduction

Neuromuscular foot deformities, particularly spastic equinovarus deformities (SED) are considered challenging to treat due to the complex underlying pathologies of muscles and nerves [1]. SED is characterized by the unopposed plantarflexion and inversion of the foot which results from improper forces acting on the hindfoot [2]. SED is amongst the most prevalent and challenging neuromuscular foot deformities. The most common etiology of SED is cerebral palsy (CP) but can also be seen in conditions such as Charcot-Marie-Tooth (CMT) disease. The prevalence of CP is 1.6 per 1000 in high income countries [3] and represents a heterogeneous group of nonprogressive neuromuscular pathologies that often manifest as spastic equinus or equinovarus deformities [4, 5]. CMT disease, a progressive neuromuscular disease with a prevalence of 15.7 per 100,000 [6], leads to bilateral equinovarus deformities due to muscle atrophy and imbalance. This is secondary to myelin sheath dysfunction [7–10].

Although rare and less commonly reported in the literature, other neuromuscular conditions that can result in SED include spina bifida, hereditary motor sensory neuropathy, myelomeningocele, and Duchenne muscular dystrophy [1]. Despite promising functional outcomes, the long-term efficacy of SPOTT remains a subject of debate due to high variability in reported failure and recurrence rates. Studies report key risk factors associated with increased failure rates including younger age at surgery (< 8 years), non-ambulatory status, quadriplegic CP subtype, and residual spasticity. Additionally, technical factors such as inadequate tendon tensioning and unaddressed bony deformities contribute to postoperative recurrence. Beyond these factors, inconsistencies in defining recurrence and failure further hinder data synthesis and clinical decision-making, making it difficult to compare outcomes across studies and develop standardized treatment protocols. The absence of a universal definition for recurrence, whether based on radiographic parameters, clinical deterioration, or the need for revision surgery, obscures long-term prognostic insights and limits the ability to refine surgical strategies.

Among various surgical techniques, the split posterior tibial tendon transfer (SPOTT) has received increasing attention as a potential intervention to address dynamic equinovarus deformities in CP patients. SPOTT involves splitting the PTT and transferring a portion of it through the interosseous membrane to the dorsum of the foot. This aims to restore balanced foot alignment while avoiding overcorrection, such as calcaneovalgus deformity.The procedure has shown improvements in gait and foot position, with some studies reporting functional improvements in affected children and adolescents [11].

However, the success of SPOTT is not without its challenges. The procedure’s failure and recurrence rates are critical considerations in evaluating its long-term efficacy. Studies have demonstrated that failure rates can be influenced by technical errors, such as improper tensioning of the transferred tendon, which has been linked to persistent deformities [12]. Furthermore, recurrence of equinovarus deformity postoperatively remains a concern, particularly in younger patients with spasticity [12]. Research indicates that children under the age of eight may experience higher rates of recurrence due to continued spasticity of the tibialis posterior tendon, which can result in the persistence of varus deformities even after successful surgery [6, 11–17].

Postoperative management plays an equally important role in improving outcomes. The use of foot orthoses and night braces in the postoperative period may help manage residual deformities and ensure continued foot function [12]. Despite some residual deformities, most patients in various studies have been able to reduce the use of orthotics following the SPOTT procedure, which indicates its success in restoring functional mobility [12].

This study aims to analyze how failure is defined in SPOTT procedures, with a focus on how these issues are managed. By evaluating outcomes of SPOTT and related tendon transfer techniques, this review seeks to contribute valuable insights into improving treatment strategies for equinovarus deformity in children with cerebral palsy. While definitions of failure remain inconsistent across studies, standardizing these criteria is essential for refining treatment strategies, improving long-term functional outcomes, and tailoring SPOTT more effectively to individual patient needs.

## Methodology

A systematic literature review was conducted across several databases, including Embase (*n* = 171), Medline (*n* = 0), and PubMed (*n* = 322), for studies published between 1995 and 2025. The search aimed to identify studies examining split posterior tibial tendon transfer (SPOTT) for spastic equinovarus foot deformity, with an emphasis on gait outcomes. A total of 792 studies were initially imported for screening. After removing 323 duplicates identified by Covidence, 463 studies remained for title and abstract screening. Of these, 447 were excluded as irrelevant, leaving 16 for further assessment. Following full-text review of 9 eligible studies, 2 were excluded based on predefined criteria, resulting in 7 studies that met inclusion criteria and were included in the final review. Studies were included based on the following criteria:

### Inclusion Criteria

- Publication Date: Studies published within the past 30 years (from 1995 onward).
- Types of studies: randomized controlled trials (RCTs), quasi-RCTs, prospective or retrospective cohort studies, case series, and case-control studies.
- Number of participants: Studies with more than 10 participants with neuromuscular disease and equinovarus deformity.
- Population: Pediatric population under the age of < 18 with a diagnosed neuromuscular disorder (e.g., cerebral palsy, stroke, Charcot-Marie-Tooth disease, traumatic brain injury) exhibiting equinovarus deformity.
- Intervention: Studies evaluating the effectiveness of SPOTT.
- Outcomes: Studies must report the recurrence rate of equinovarus deformity post-SPOTT, with a minimum follow-up of 24 months on average, or provide instrumented gait analysis with a mean follow-up of over 12 months.

### Exclusion Criteria

- Types of studies: Studies that do not report subgroup data for eligible patients and for which author contact was unsuccessful.
- Number of participants: Studies with fewer than 10 participants specifically with neuromuscular disease and equinovarus deformity.
- Population: Studies not involving patients with both a neuromuscular disorder and equinovarus deformity.
- Intervention: Studies not evaluating SPOTT or evaluating only other procedures (e.g., anterior tibialis tendon transfer).
- Outcomes: Studies that do not report recurrence rates post-SPOTT or do not provide instrumented gait analysis with appropriate follow-up duration.

**Fig. 1:**
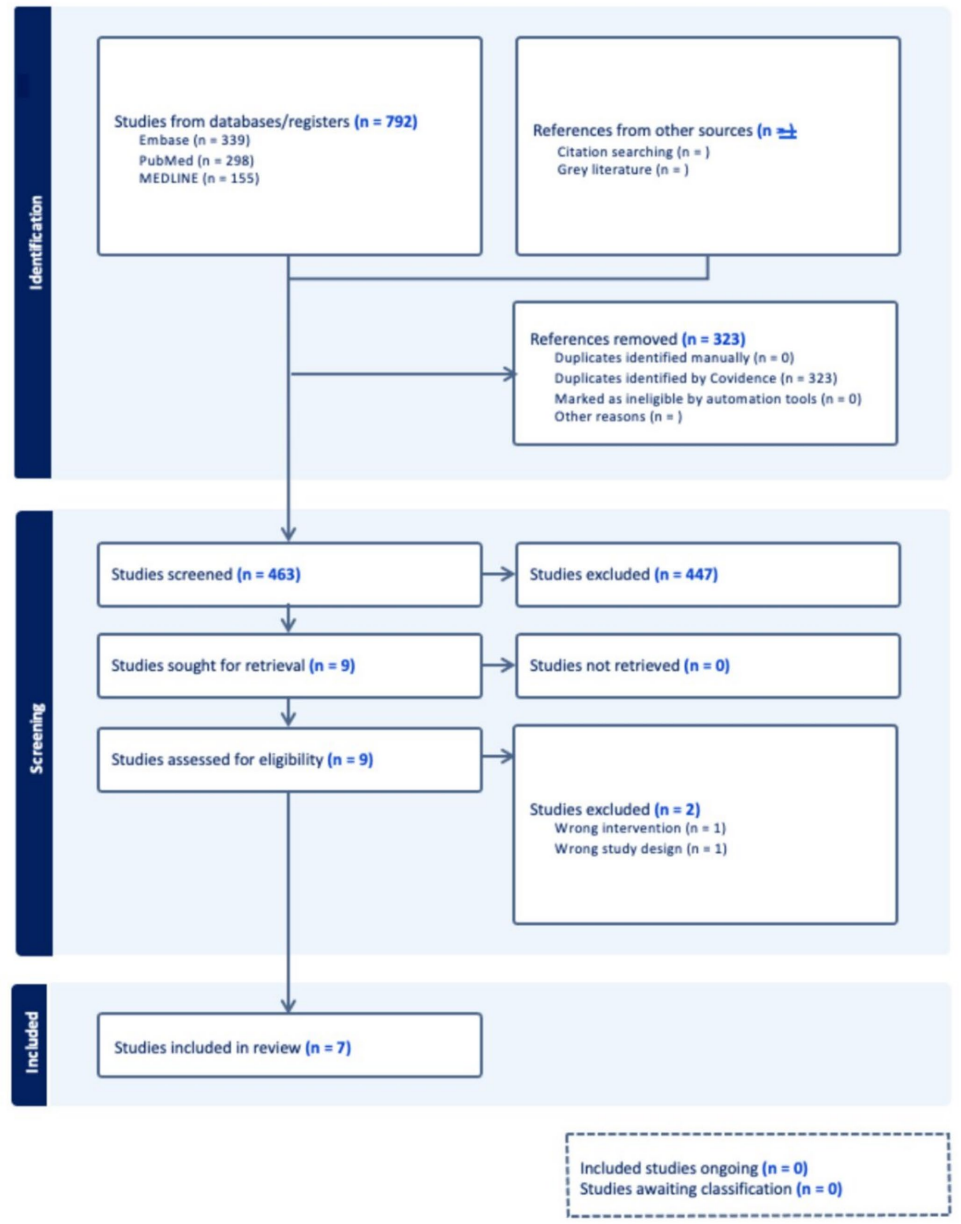
PRISMA flow diagram outlining the process of study identification, screening, eligibility assessment, and inclusion

Initial screening was based on titles and abstracts. Full texts were obtained for studies meeting initial screening criteria, and further assessed for eligibility. The number of studies screened, assessed for eligibility, and included in the review are detailed in a PRISMA flow diagram as seen in Fig. 1.

The methodological quality and relevance of the included studies were assessed during full-text review using structured reviewer consensus within Covidence. Each study was reviewed for risk of bias, applicability to the research question, and methodological rigor. A total of 9 studies were assessed for quality and evidence synthesis (Fig. 1).

## Results

### Population Characteristics

The patient populations were consistently limited to ambulant children with CP presenting with flexible spastic equinovarus deformity across the seven included studies. Patients who were non-ambulant and with fixed bony deformities were uniformly excluded. This is consistent with the indication of SPOTT for correctable soft tissue imbalance rather than rigid skeletal malalignment.

The total number of feet treated per study ranged from 13 to 146, with most studies evaluating cohorts between 25 and 140 feet. Mean age at surgery varied from 5.5 to 11 years. Follow-up durations ranged from 2.4 years to 10 years, with only three studies providing long-term data (> 7 years).

Sex distribution showed male predominance overall, although ratios varied. While some studies reported near-equal male-to-female representation, others demonstrated skewed distributions in either direction. Complete demographic breakdowns are summarized in Table 1.

**Table 1:** Demographic and surgicalcharacteristics of included studies

Study	Year	Country	Study Design	Total Feet	Mean Age at Surgery (yrs)	Age Variance (yrs)	Mean Follow-up Duration (yrs)	Follow-Up Variance (yrs)	Overall Failure Count (feet)
Ahmed et al.	2011	Pakistan	retrospective case series.	17	8	Range: 6–10	3.5	Range: 2–4	2
Aleksic et al.	2020	Serbia	retrospective cohort study	146	11	IQR: 6–14.25	8	IQR: 6–11	29
Chang et al.	2002	United States of America	longitudinal cohort study	88	9.4	Range: 2.7–16.9	7.3	Range: 2.6–13.8	39
Dussa et al.	2021	Germany	retrospective comparative study.	13	NA	Range: 6.0–22.0 years	2.4	SD: ±0.8 years	4
Mulier et al.	1995	Belgium	retrospective cohort study	21	5.5	Range: 4–9	3	Range: 2.9–6.0 yrs	2
Sayan et al.	2024	United States of America	retrospective analysis	44	9.8	SD: 3.5	3.5	SD: 1.6	22
Vlachou et al.	2010	Greece	retrospective case series	38	10.8	Range: 6–17	10	Range: 4–14	4

Summary of demographic and surgical characteristics of included studies, detailing country, study design, patient demographics, follow-up duration, and overall failure counts by feet

### Cerebral Palsy Subtypes

The following studies examined showed variability in the distribution of cerebral palsy (CP) subtypes; however, consistent trends were reported. Unless specified, the following percentages correspond to the proportion of patients rather than their feet. The only exception is Vlachou et al. [11] since the reported CP subtypes are based on the number of feet rather than the patients. Essentially, Vlachou et al. were excluded from Table 2 due to not converting the data to patient-level proportions. Spastic hemiplegia was the most prevalent subtype across nearly all studies, with a prevalence ranging from 69.2% (9/13) in Ahmed [6] to 75% (30/40) in Sayan [7]. Intermediate values were reported by Aleksic (2020) at 52.4% (65/124) and Mulier [14] at 59% (10/17). Spastic diplegia was the second most prevalent subtype. For instance, Ahmed reported 23.1% (3/13), 30.6% (38/124) in Aleksic, 12% (2/17) in Mullier, and 22.5% (9/40) in Sayan. Spastic quadriplegia was less frequently reported. For instance, Ahmed noted 7.77% (1/13), 12.1% (15/124) in Aleksic, and 29% (5/17) in Mulier. However, Sayan and Dussa did not report quadriplegia as a subtype. Finally, other subtypes were observed even less. Aleksic identified 4.8% (6/124) of patients as having spastic triplegia and 17.7% (22/124) as having spastic paraplegia. Sayan identified 2.5% (1/40) with triplegia but did not report paraplegia. Dussa [15] did not stratify patients by CP subtype.

**Table 2:** Reported frequencies of spastic cerebral palsy subtypes across studies

CP Subtype	Ahmed (2011)	Aleksic (2020)	Dussa (2021)	Mulier (1995)	Sayan (2024)
Spastic hemiplegia	69.2% (9/13)	52.4% (65/124)	Not reported	59% (10/17)	75% (30/40)
Spastic diplegia	23.1% (3/13)	30.6% (38/124)	Not reported	12% (2/17)	22.5% (9/40)
Spastic paraplegia	Not reported	17.7% (22/124)	Not reported	Not reported	Not reported
Spastic Triplegia	Not reported	4.8% (6/124)	Not reported	Not reported	2.5% (1/40)
Spastic Quadriplegia	7.7% (1/13)	12.1% (15/124)	Not reported	29% (5/17)	Not reported

Reported frequencies of spastic cerebral palsy subtypes across included studies, highlighting variability in subtype distribution and gaps in reported data

These Findings highlight the predominance of spastic hemiplegia and diplegia among surgical candidates for SPOTT. In contrast, quadriplegic and triplegic subtypes are generally associated with poorer outcomes. Essentially, the following trends highlight the importance of subtype-specific surgical planning and thorough preoperative functional assessment. A complete summary of CP subtype distribution by study is presented in Table 2.

An additional limitation is that Chang et al. [1] included patients undergoing both SPOTT and Z-lengthening procedures without reporting CP subtype by surgical group. Therefore, their data were excluded from Table 2, although subtype percentages were mentioned in the text.

### Surgical Techniques Practiced

The reviewed studies investigated variations of the SPOTT technique for managing spastic equinovarus deformity in cerebral palsy. Aleksić et al. (2020) compared the standard SPOTT (per Green et al.) with a modified version incorporating Z-plasty elongation of the medial tibialis posterior (TP) tendon and a longitudinal split for improved balance, yielding superior outcomes. Chang et al. [1] utilized the standard SPOTT with Z-lengthening of the posterior tibialis. Mulier et al. [14] employed an anterior interosseous SPOTT, routing the tendon through the interosseous membrane to the dorsum, combined with Achilles tendon lengthening. Vlachou et al. [11] followed the Green et al. technique, making four incisions and transferring the tendon to the peroneus brevis via a posterior route. Sayan et al. [7] also utilized the Green method, detaching the plantar half of the tendon from the navicular, passing it posteriorly around the tibia and fibula, and securing it to the peroneus brevis via Pulvertaft weave. Ahmed et al. [6] employed a technique similar to Mulier, splitting the tibialis posterior and transferring the anterior half through the interosseous membrane to the lateral cuneiform, with concomitant percutaneous tendo-Achilles lengthening in all cases. Dussa et al. [15] used a combined SPOTT and SPLATT approach for flexible varus deformity, performing a posterior tibialis split transfer via the interosseous route to the peroneus brevis and anchoring with a Pulvertaft weave, as well as a split anterior tibialis transfer to the cuboid. These technique variations reflect ongoing refinements to improve tendon balance, foot progression angle, and long-term correction in this complex population.

### Indications/Goals for Surgery

The rationale behind conducting SPOTT procedures in quadriplegic patients is focused on preventing complications rather than improving gait. Mulier et al. emphasized that SPOTT in quadriplegic patients helps prevent skin breakdown and pressure ulcers by correcting persistent equinovarus positioning, thereby improving tolerance for bracing and wheelchair use. Chang et al. further adds to this rationale by highlighting the benefits of facilitating standing, assisted walking, preventing contracture progression, and improving wheelchair positioning. However, Vlachou does not provide any explicit justification for including quadriplegic patients and their study reports high failure rates without analysis. Aleksić et al. reported similar findings, raising concern over outcomes and benefits in this subgroup. Overall, while SPOTT can offer improvements in positioning and caregiving tolerance, the decision to operate should be individualized, with goals centered on comfort rather than ambulation.

### Initial Management

Pre-operative planning for SPOTT prioritizes the evaluation of flexible deformities and confirmation of ankle dorsiflexion. Patients with fixed bony deformities should undergo corrective bone procedures prior to tendon transfer to prevent persistent varus alignment. Common adjunctive procedures include plantar soft tissue release, aimed at lengthening the shortened base of the foot [11]. Additionally, modified surgical techniques, such as Z-plasty elongation of the posterior tibial tendon, have been employed to improve foot positioning and reduce recurrence risk [11].

Post-operative protocols also varied among studies. Sayan [7] implemented a structured plan involving three weeks of non–weight-bearing casting, followed by three weeks of weight-bearing casting, and then transition to a short ankle-foot orthosis (SAFO). This was paired with an intensive rehabilitation program focused on range of motion and strength. Surgical outcomes were assessed after one year using motion analysis to guide further bracing decisions [17].

Similarly, Ahmed [6] used an above-knee plaster cast with the ankle in a neutral position, permitting touchdown weight-bearing after three weeks. At six weeks, patients transitioned to an ankle-foot orthosis (AFO), which was continued for six months. Patients with favorable outcomes were advised to continue night splinting, while those with unsatisfactory results were considered for further surgical intervention, including calcaneocuboid fusion 18 months postoperatively [16].

### Overall Recurrence and Failure

Across the reviewed studies, failure and recurrence rates following SPOTT procedures varied significantly, often reflecting inconsistencies in how these outcomes were defined and reported. While some studies provided explicit criteria for failure or recurrence, others relied on indirect or inferred definitions. A summary of the failure definitions and corresponding failure rates across all included studies is presented in Table 3. Aleksic et al. (2020) reported a failure rate of 19.9% (29/146 feet), defining failure as either a poor clinical outcome or the need for revision surgery, including triple arthrodesis. The highest failure rates in their cohort were seen in patients with GMFCS level IV (90.9%) and spastic quadriplegia (86.7%), compared to lower rates in GMFCS III (30.2%) and spastic diplegia (31.0%). Chang et al. [1] used a more specific definition—postoperative hindfoot deformity ≥ 10° in varus or valgus or the need for reoperation—and reported a failure rate of 44.3% (39/88 feet). Failures were more common in younger patients (< 8 years), non-ambulators, and those with quadriplegia (75% failure in 16 feet). Dussa [15] defined failure as either overcorrection (hindfoot valgus/flatfoot) or undercorrection (persistent varus), with a failure rate of 30.8% (4/13 feet). Sayan [7] inferred failure from postoperative foot progression angle falling outside the desired range (0–10° of external rotation) and reported a 50% failure rate (22/44 feet). Failures were linked to inconsistent surgical technique, untreated deformities, and tendon retraction. In contrast, Ahmed [6] and Mulier et al. [14] reported lower failure rates of 11.8% (2/17) and 9.5% (2/21), respectively, though both studies lacked explicit failure definitions. In these cases, failure was inferred from persistent deformity or technical error such as poor tendon tensioning or misidentification of the dominant deforming muscle. Vlachou et al. [11] similarly lacked a formal definition but reported a 10.5% failure rate (4/38 feet), with failure generally interpreted as recurrence requiring further intervention.

**Table 3:** Definitions of recurrence and failure across included studies

Study	Definition of Recurrence Used	Definition of Failure Used	Overall Failure Count (feet)
Ahmed et al. (2011)	Recurrence was not explicitly defined but was inferred as the return of the equinovarus deformity following surgery.	Failure was not explicitly defined but was interpreted as the persistence or reappearance of the deformity, consistent with a poor surgical outcome.	2/17 (11.8%)
Aleksic et al. (2020)	Recurrence was defined as the reappearance of the equinovarus deformity or overcorrection into valgus, regardless of whether revision surgery was required.	Failure included cases classified as poor outcomes and those requiring revision surgery, such as a triple arthrodesis.	29/146 (19.9%)
Chang et al. (2002)	Recurrence was defined as a return of the deformity following initial surgical correction.	Failure was defined as a postoperative hindfoot deformity of ≥ 10° in varus or valgus, or the need for an additional surgical procedure.	39/88 (44.3%)
Dussa 2021	Recurrence was indirectly defined as the persistence or return of the varus foot deformity following surgery.	Failure was defined as either overcorrection (hindfoot valgus/flatfoot) or undercorrection (persistent varus deformity), and included cases of recurrence.	4/13 (30.8%)
Mulier et al. (1995)	Recurrence was not explicitly defined but was inferred as the return of equinovarus deformity postoperatively, particularly persistent varus alignment.	Failure was not explicitly defined but was inferred as an unsuccessful correction due to surgical or technical error, such as tendon loosening or improper tensioning, resulting in persistent or worsening deformity.	2/21 (9.5%)
Sayan et al. (2024)	Recurrence was not explicitly defined in this study, but was implied as a return of equinovarus deformity or failure to maintain correction in foot progression angle post-operatively.	Failure was not directly defined, but functionally indicated by a post-operative foot progression angle falling outside the target range of 0–10° of external rotation, including both under- and over-correction.	22/44 (50%)
Vlachou et al. (2010)	Recurrence was not explicitly defined but was inferred as the return of equinovarus deformity following SPOTT, necessitating revision surgery such as calcaneocuboid fusion.	Failure was not explicitly defined but was inferred as persistent or recurrent severe deformity requiring additional bracing or surgical intervention due to pain, callosities, or difficulty with weight-bearing.	4/38 (10.5%)

This table summarizes how each study defined “recurrence” and “failure” following split posterior tibial tendon transfer (SPOTT) in children with cerebral palsy. Notably, many studies lacked standardized or explicit definitions. Failure counts are reported as the number of feet meeting the respective study’s criteria, out of the total number assessed

Overall, the definitions of both recurrence and failure across the literature remain heterogeneous. Some studies used radiographic or angular thresholds, while others relied on clinical judgment, revision surgery, or the presence of complications such as pain or callosities. This inconsistency poses a significant barrier to data synthesis and cross-study comparisons and underscores the need for a standardized definition to guide both clinical evaluation and research reporting (see Table 3).

### Patient-Specific Risk Factors for Failure

Several patient-specific factors have been consistently linked with higher failure and recurrence rates following split posterior tibialis tendon transfer (SPOTT) in children with cerebral palsy, however, the strength of statistical evidence varies across studies, as shown in Table 4. It was reported that patients under 8 years old were the most frequently identified risk factor. This link was addressed in at least four studies [6, 13, 15, 20]. Chang et al. [1] [6] found a significant increase in failure rates in children under 8. Essentially, Chang et al. [1] [6] reported a 61% failure rate (*p* < 0.05) in this group. In addition, Aleksic et al. (2020) [13] and Vlachou et al. (2010) [11], came to a similar conclusion. They reported higher recurrence rates among younger patients, however neither conducted formal statistical testing. Furthermore, Mulier et al. [14] also noted age-related recurrence but did not provide a statistical analysis of their data.

**Table 4:** Summary of Patient-Specific and surgical risk factors associated with failure of split posterior tibialis tendon transfer (SPOTT) in children with cerebral palsy

Study	Risk Factor	Association	Statistical Evidence
Chang et al. (2002)	Age < 8 years	61% failure rate	Yes (*p* < 0.05)
Aleksic et al. (2020)	Age < 8 years	Higher recurrence	No
Vlachou et al. (2010)	Age < 8 years	Higher recurrence	No
Mulier et al. (1995)	Age < 8 years and > 16 years	Higher recurrence and failure	No
Chang et al. (2002)	CP Severity	Failure: Hemiplegia 23%, Diplegia 52%, Quadriplegia 66^	Yes (*p* = 0.004)
Vlachou et al. (2010)	CP Severity	Worse in diplegic/quadriplegic than hemiplegic	Yes (*p* = 0.005)
Chang et al. (2002)	Poor ambulation	Failure: 79% (non-ambulatory) vs. 23% (ambulatory)	Yes (*p* < 0.01)
Aleksic et al. (2020)	Poor ambulation	Significantly higher failure rate	Yes (*p* < 0.01)
Ahmed et al. (2011)	Severe deformity	Poor correction outcomes	No
Mulier et al. (1995)	Limited hindfoot mobility	Restricted postoperative correction	No
Vlachou et al. (2010)	Proximal malalignment	Residual deformity	No
Aleksic et al. (2020)	Tendon tensioning errors	Under/Over-correction	No
Mulier et al. (1995)	Tendon tensioning errors	Under/Over-correction	No
Sayan et al. (2024)	Tendon retraction /lengthening issues	Recurrence	No
Vlachou et al. (2010)	Tendon retraction	Recurrence	No
Sayan et al. (2024)	Inter-surgeon variability	Inconsistent outcomes	No
Ahmed et al. (2011)	Misidentification of dominant muscle	Under-correction	No

Synthesizes the findings from multiple studies assessing the impact of demographic, neurological, functional, and technical factors on SPOTT outcomes. Reported associations are listed alongside corresponding statistical significance where available

*Abbreviations* CP, cerebral palsy

*Note* Only associations explicitly discussed in the referenced studies are included. “No” under statistical evidence indicates that the study did not report formal statistical testing to support the association

In contrast, older patients over 16 years old, were found to have a higher failure rate, potentially due to the reduced joint adaptability and the presence of more rigid deformities. This finding was reported by Mulier et al. [14], although no statistical evidence was provided.

Additionally, the cerebral palsy subtypes were another major factor in the clinical outcome. For instance, Chang et al. [1] [6] reported increasing failure rates were linked with neurological severity: 23% in spastic hemiplegia, 52% in diplegia, and 66% in quadriplegia, with a statistically significant difference (*p* = 0.004). Likewise, Vlachou et al. (2010) [11] reported significantly poor outcomes in diplegic and quadriplegic patients compared to hemiplegics (chi-square, *p* = 0.005), thereby highlighting the link between CP severity and SPOTT outcomes.

The preoperative ambulatory function has been shown to be a major factor in surgical outcomes. For instance, both Chang et al. [6] and Aleksic et al. [13] reported that patients with limited or no ambulation had significantly higher failure rates, with values reaching up to 79% compared to 23% in ambulatory patients (*p* < 0.01). Essentially, the following findings support the notion of baseline functional status and serve as a valuable prognostic indicator.

Other patient-specific risk factors have been reported with limited statistical data. Ahmed et al. [16] identified severe preoperative deformity— Characterized by equinus greater than 25° or varus greater than 20°—along with marked Achilles tendon contracture as a significant factor in poor correction outcomes; however, they do not provide statistical data to corroborate these findings. Mulier et al. [14] noted that limited preoperative hindfoot mobility appeared to restrict postoperative correction, while Vlachou et al. [11] highlighted the necessity of addressing proximal alignment issues such as femoral or tibial torsion, to limit residual deformity. Nevertheless, these factors were discussed qualitatively, without statistical analysis, making it difficult to gauge their impact.

In conclusion, younger age, greater severity of cerebral palsy (CP), and poor baseline ambulation have been statistically associated with poor outcomes of SPOTT. However, numerous other risk factors remain within the studies. This highlights the importance of future multivariate analyses to better quantify their relative influence.

### Surgical Risk Factors for Failure

The surgical technique represents a crucial factor in influencing the success of SPOTT, as shown in Table 4. The most commonly reported surgical technique complication was inadequate tensioning of the tendon, which may have caused an under-correction or overcorrection of the deformity. This complication was reported in three studies [13–15]; however, none of the following studies conducted statistical analysis that linked tensioning errors with surgical outcomes.

Another contributing factor to under-correction is the misidentification of the dominant muscle responsible for the deformity, especially in cases involving mixed or atypical deformity patterns [16]. However, no statistical evidence was provided.

Additional factors contributing to recurrences, such as tendon retraction and insufficient tendon lengthening were reported in both Sayan [7] and Vlachou [11]. Sayan et al. further highlighted that inter-surgeon variability in technique was a major factor in inconsistent surgical outcomes. However, statistical evidence was not provided.

In conclusion, the modes of failure varied across studies such as under-correction, over corrections (into hindfoot valgus or flatfoot), persistent varus deformity, and the need for revision surgeries such as triple arthrodesis of calcaneocuboid fusion. Despite these findings, the majority of the reports addressing surgical risk factors remain largely descriptive, with very few studies providing statistical evidence for technical complications to surgical failures.

### Management of Failure

Various strategies have been proposed for addressing failure or recurrence following SPOTT. These approaches include continued bracing, revision surgeries and additional procedures individualized to the specific nature of the recurrence. Aleksic et al. emphasized that revision surgeries, specifically triple arthrodesis, are frequently performed on patients who developed recurrent equinovarus or overcorrected into valgus deformity.

In addition, Vlachou highlighted the importance of bracing and additional corrective surgeries, such as calcaneocuboid fusion, for severe recurrences. Furthermore, Chang recommended repeating tendon lengthening for cases of recurrent varus, while patients presenting with significant valgus deformities were managed with an ostomy or subtalar arthrodesis. Several studies have highlighted that the management of failure typically involves a combination of prolonged bracing, surgical revisions, and targeted procedures, depending on the deformity.

### Study-Level Bias

Bias was consistently identified in all studies as a result of retrospective methodologies, absence of blinding, and irregular outcome reporting [6, 11, 13, 14, 17]. Selection bias was introduced due to non-random patient inclusion, with additional distortion caused by excluding non-ambulatory patients or individuals with fixed deformities [11, 14, 17]. Attrition bias was especially evident in Vlachou, where fewer than 50% of qualified patients were present at the final follow-up without analyzing those who were lost [11], and in Aleksic, which documented 8% of missing data with no imputation [13]. In Sayan, the omission of patients undergoing bony rotational procedures might have skewed the sample towards simpler deformities [17]. Performance bias might be present in all studies because of various surgeons and differing perioperative protocols, which were neither standardized nor reported comprehensively. Detection bias was probable in Mulier and Vlachou, as they predominantly depended on unblinded, subjective clinical evaluations lacking objective measures [11, 14]. Reporting bias was apparent in all studies, particularly with the consistent lack of patient-reported outcome measures [11, 14, 17], hindering the assessment of functional benefits from the patient’s viewpoint.

## Discussion

The most important finding of this review is the substantial inconsistency in how surgical failure and recurrence are defined across studies evaluating SPOTT in children with cerebral palsy. Definitions ranged from quantitative angular deformities to qualitative clinical judgments or the necessity of revision surgery, with several studies offering no explicit criteria at all. This definitional heterogeneity significantly impairs the ability to compare outcomes, synthesize data, and draw evidence-based conclusions. As summarized in Table 3, failure definitions varied from specific angular thresholds (e.g., hindfoot deformity ≥ 10° in Chang et al.) to more subjective indicators such as recurrence of symptoms, need for reoperation, or persistent gait issues.

Reported failure rates reflected this variability. They ranged from as low as 9.5% (2/21) in Mulier et al. to as high as 50% (22/44) in Sayan et al., with intermediate rates in studies like Aleksic et al. (19.9%), Chang et al. (44.3%), and Dussa (30.8%). Studies using broader or more functionally sensitive definitions, such as Chang et al., consistently reported higher failure rates, whereas those using narrower surgical thresholds, such as Vlachou et al., showed lower recurrence but may have underrecognized clinically significant impairments.

Multiple risk factors for failure emerged, encompassing both patient-specific and surgical elements. Younger age (< 8 years) was the most commonly reported predictor of recurrence, attributed to persistent spasticity and musculoskeletal immaturity. Non-ambulatory status and more severe CP subtypes (particularly quadriplegia) were also strongly associated with poor outcomes. On the surgical side, technical issues such as inadequate tendon tensioning, failure to identify the dominant deforming muscle, and lack of standardized postoperative bracing protocols were frequently implicated in recurrence or overcorrection. Despite the prevalence of these observations, only a minority of studies performed statistical testing to validate these associations, limiting their generalizability.

These findings underscore the critical need for a standardized recurrence grading system. Without it, interpretation of surgical outcomes remains ambiguous, and long-term efficacy is difficult to assess. If such a system had been in place, a more consistent understanding of SPOTT performance across patient subgroups and time points could have been achieved.

This review also adds perspective on long-term recurrence, an area that remains under-reported in the literature. Most prior studies focused on short-term outcomes (1–3 years), whereas several of the included studies in this review suggested that recurrence often emerges beyond that window. Hemiplegic patients consistently demonstrated the lowest failure rates, whereas quadriplegic and triplegic patients—when included—had the highest. These trends align with the known neuromuscular severity gradient of CP subtypes and suggest that baseline motor impairment substantially influences surgical durability.

The choice of surgical method (e.g., Z-lengthening, intramuscular lengthening, or tendon transfer) did not significantly affect recurrence rates across studies. However, technical aspects of the procedure, particularly intraoperative tendon tensioning, appeared to be a common contributor to undercorrection and overcorrection. Multiple studies reported improved outcomes with the use of postoperative bracing for a minimum of six months, and in more severely involved patients, several authors recommended delaying surgery until after age 8 to reduce recurrence risk [6, 14]. Comprehensive preoperative planning—including gait analysis, EMG, and ROM assessments—was emphasized as essential for patient selection and surgical planning [11]. Long-term success ultimately depends on intraoperative precision, post-operative rehabilitation, and a multidisciplinary approach to care [11, 17].

### Limitations and Methodological Considerations

This review has a few notable limitations. For instance, all included studies were retrospective and exhibited variability in their design, surgical techniques, and outcomes. In addition, the majority of the studies did not display control groups, which increased the risk of bias. Furthermore, the small sample sizes and the limited follow-up beyond skeletal maturity weakened the reliability of the pooled conclusions. Finally, the majority of the studies did not include patient-reported outcome measures (PROMs) but instead relied on subjective clinical assessment or static radiographs; with only one study using an instrumented gait analysis.

A prominent challenge in this field was the inconsistency in definitions for surgical failure and recurrence. The term variation ranged from radiographic parameters to clinical deteriorations. Essentially, these inconsistencies made it challenging to compare results among other studies. In addition, angular threshold and surgical assessments differed significantly among studies, which led to substantial variations in failed outcomes. Finally, the documentation of the simultaneous procedures was often not reported clearly, which introduced possible confounding factors that could influence the results.

To conclude, addressing the limitations in this review process is essential. Despite the comprehensive search across major databases, the small number of eligible studies (*n* = 7) restricted the ability to conduct a more detailed subgroup analysis. In addition, the inconsistencies in reporting formats prevented meta-analysis. This study relied on reported data from the studies, without access to patient-level information, which may affect the outcome. In conclusion, these limitations should be addressed when interpreting the results and for future investigations.

As observed in multiple studies, the rates of recurrence and failure significantly rely on the definitions provided by each author. Wider criteria, like those applied by Chang et al., generally reported higher recurrence rates, whereas narrower definitions, as seen in Vlachou et al. - show lower figures but may overlook functional impairments that do not necessitate surgery.

The absence of standardized recurrence metrics, previously noted as a limitation—continues to impact meaningful cross-study comparisons and meta-analytic synthesis. This study identifies key limitations in prior literature:

- *Subjectivity in Defining Success vs. Recurrence*: Most studies rely on subjective clinical grading systems with vague or inconsistent definitions for success, recurrence, and failure [6, 11, 13, 14], limiting comparability.
- Lack of Long-Term Follow-Up: Several papers report outcomes within 1–3 years [14, 17], yet published findings suggest recurrence may emerge beyond that window, underscoring the need for studies with follow-up beyond skeletal maturity [11, 13].
- *Inconsistent Use of Radiographic and Functional Assessments*: Only one study utilized instrumented gait analysis [17], while others relied primarily on static radiographs or unblinded clinical observation, missing dynamic and functional gait changes critical to patient mobility [11, 14].
- *Absence of Patient-Reported Outcomes*: No studies incorporated patient-reported outcome measures (PROMs), making it impossible to assess impact from the patient or caregiver perspective, an increasingly essential metric in modern surgical evaluation [11, 14, 17].

### Recommendations for Future Research

Future research should place standardized radiographic, functional, and patient-reported outcomes to enhance the comparability and clinical applicability of results. Essential suggestions consist of:

- *Regular Follow-Up at 2, 5, and 10 Years*: Established intervals for evaluation will allow for reliable monitoring of recurrence rates and functional durability over time.
- *Combining Functional Metrics*: Future studies should combine radiographic evaluations with quantitative gait analysis, pedobarographic pressure assessments, and validated (PROMs) to encompass both biomechanical and experiential outcomes.
- *Standardized Definitions for Recurrence and Failure*: Well-defined, widely accepted definitions are crucial for enabling comparisons between studies and the analysis of combined data.
- *Managed Accounting for Simultaneous Procedures*: Research must either manage or categorize by concurrent surgical actions to separate SPOTT-related impacts.
- *Collaboration Across Multiple Centers for Validation*: Studies involving multiple institutions may help validate criteria for recurrence grading, outcome thresholds, and surgical protocols across diverse patient populations.
- *Comprehensive Surgical Documentation*: Procedural aspects like tendon tensioning techniques and fixation approaches must be clearly documented due to their proven influence on results and consistency.

## Conclusion

Overall, SPOTT remains a potentially viable option for correcting dynamic equinovarus deformity in children with cerebral palsy, but long-term outcomes are highly variable due to multiple patient and surgery specific risk factors. Recurrence and failure are consistently associated with younger age at surgery (< 8 years), quadriplegic subtype, non-ambulatory status, and severe preoperative deformities. Technical contributors include poor tendon tensioning, untreated bony malalignment, residual spasticity, and misidentification of the dominant deforming muscle during preoperative planning. Surgical technique variability, delayed or inconsistent rehabilitation, proper preoperative assessments, and insufficient postoperative bracing further increase the risk of recurrence.

Modified SPOTT techniques such as Z-lengthening, anterior routing, and combined soft-tissue procedures have shown improved outcomes in some cohorts; however, no single technique has consistently prevented recurrence. Long-term success is more likely in patients over age 8, with less severe deformities, appropriate muscle targeting, and structured bracing protocols.

Synthesizing reliable evidence remains difficult as the lack of standardized definitions for recurrence and failure continues to be a major barrier. Studies varied widely: some used radiographic angles, others surgical revision, and many failed to specify criteria at all. The inconsistencies in these definitions may hinder the development of future research.

In order to enhance future outcome reporting, standardized definitions must be used to enable consistent evaluation of SPOTT procedures in this patient population moving forward. Other factors must also be taken into careful consideration and improved in order to reduce failure rates and improve functional outcomes such as: individualised preoperative assessment, technical precision and surgical expertise, and postoperative care.

## Key References

1. Chang CH, Albarracin JP, Lipton GE, Miller F. Long-term follow-up of surgery for equinovarus foot deformity in children with cerebral palsy. *J Pediatr Orthop*. 2002;22:792–799.

- ○ This study demonstrates long-term follow up data important for understanding the effectiveness and limitations of surgical interventions for equinovarus foot deformities in children with CP. The correlation between risk factors such as age can influence surgical outcomes and recurrence which is essential for guiding proper clinical decisions.

2. Vlachou, M., Beris, A., Dimitriadis, D. (2010). Split tibialis posterior tendon transfer for correction of spastic equinovarus hindfoot deformity. *Acta Orthopaedica Belgica*, 76(5), 651–657​.

- ○ This article provides insights on the role of SPOTT for correcting spastic equinovarus foot deformities and insights into the effectiveness and long-term outcomes of the procedure.

3. Aleksić, M., Baščarević, Z., Stevanović, V., Rakočević, J., Baljozović, A., Čobeljić, G. (2019). Modified split tendon transfer of posterior tibialis muscle in the treatment of spastic equinovarus foot deformity: long-term results and comparison with the standard procedure. *International Orthopaedics*, 10.1007/s00264-019-04443-6​.

- ○ This study highlights the modified SPOTT technique, demonstrating superior long-term outcomes compared to the traditional procedure. The findings provide value into optimizing surgical approaches for effective correction of equinovarus foot deformities.

4. Mulier, T., Moens, P., Molenaers, G., Spaepen, D., Dereymaeker, G., Fabry, G. (1995). Split posterior tibial tendon transfer through the interosseus membrane in spastic equinovarus deformity. *Foot & Ankle International*, 16(12), 754–759.

- ○ This study provides early understanding into clinical applications of the SPOTT technique and associated outcomes highlighting initial effectiveness of this technique in treating equinovarus foot deformities.

5. Chakravarthy U Dussa, Harald Böhm, Leonhard Döderlein, Albert Fujak, Treatment of spastic varus/ equinovarus foot with split-tendon transfers in cerebral palsy: How does it affect the hindfoot motion?, Gait & Posture, Volume 92, 2022, Pages 343–350, ISSN 0966–6362,10.1016/j.gaitpost.2021.10.042.

- ○ This study provides significant kinematic data on how the procedure influences foot mechanics and hindfoot mobility, offering insights into the effectiveness of SPOTT for improving gait and overall functional outcomes in this patient group.

6. Ahmed, Gulzar & Shaikh, Bilal Fazal & Shaikh, & Memon, Abdul. (2011). Surgical treatment of equinovarus deformity of foot in children with cerebral palsy. Medical Channel. 17. 3-2011.

- ○ Comprehensive long-term follow up data is provided on surgical techniques correcting spastic equinovarus deformities, providing essential information on failure and recurrence rates. The findings highlight key factors influencing postoperative outcomes and durability of surgical corrections in patients with CP.

7. De Sayan, Austin Skinner, Alex Tagawa, Wade Coomer, Jason Koerner, Lori Silveira, James Carollo, Jason Rhodes, Effect of split posterior tibialis tendon transfer on foot progression angle in children with cerebral palsy, The Foot, Volume 59, 2024, 102,087, ISSN 0958–2592, 10.1016/j.foot.2024.102087.

- ○ The study provides valuable quantitative data on the effect of the procedure on gait mechanics, helping to refine surgical strategies and assess the functional impact of SPOTT in improving foot alignment and mobility post-surgery.

## Data Availability

No datasets were generated or analysed during the current study.
